# Postoperative Megarectum in an Adult Patient with Imperforate Anus and Rectourethral Fistula

**DOI:** 10.1155/2015/613926

**Published:** 2015-03-15

**Authors:** Yoshifumi Nakayama, Toshihito Uehara, Masaki Akiyama, Noritaka Minagawa, Takayuki Torigoe, Naohiro Fujimoto

**Affiliations:** ^1^Department of Surgery 1, School of Medicine, University of Occupational and Environmental Health, 1-1 Iseigaoka, Yahata-nishi-ku, Kitakyushu 807-8555, Japan; ^2^Department of Gastroenterological and General Surgery, Wakamatsu Hospital of University of Occupational and Environmental Health, 1-17-1 Hamamachi, Wakamatsu-ku, Kitakyushu 808-0024, Japan; ^3^Department of Urology, School of Medicine, University of Occupational and Environmental Health, 1-1 Iseigaoka, Yahata-nishi-ku, Kitakyushu 807-8555, Japan

## Abstract

This report presents a surgical case of postoperative megarectum in an adult patient with imperforate anus/anorectal malformations. A 71-year-old Japanese male presented with a mass in the lower abdomen which was 15 × 12 × 8 cm in diameter, edema in the right lower extremity, and frequent urination. He had undergone sigmoid loop colostomy for an imperforate anus as a newborn infant. At 28 years of age, the sigmoid loop colostomy was changed to sigmoid divided colostomy in the left lower abdomen. Computed tomography revealed a large cystic mass in the lower abdomen. Retrograde urethrography indicated a rectourethral fistula and megarectum with stones. A small laparotomy incision was created in the right lower abdomen, and the wall of the megarectum was identified. Approximately 2,300 mL of gray muddy fluid was identified and drained. A mucous fistula of the upper rectum was created in the right lower abdomen. This is an extremely rare case of postoperative megarectum in an adult patient with an imperforate anus and rectourethral fistula.

## 1. Introduction

Imperforate anus/anorectal malformations occur in approximately 1 in 5000 births [1, 2]. This rare anomaly is usually detected and repaired by pediatric surgeons during the neonatal period or early infancy. Imperforate anus is conventionally classified into three main types [3, 4]. In patients with a high imperforate anus, the bowel ends above the pelvic floor, typically with a fistula to the prostatic urethra in males or the posterior vaginal fornix in females. In patients with an intermediate imperforate anus, the bowel traverses the pelvic floor with its crucial puborectalis sling but fails to migrate back to the normal anal site. A low imperforate anus involves a covered anus or anal stenosis [3].

Megarectum is a rare condition defined as follows. Patients with idiopathic megarectum have a bowel diameter greater than 6.5 cm at the pelvic brim on a lateral X-ray of the abdomen [5]. The* Principles of Surgery* textbook describes a rectum that can hold more than 1,500 cc of fluid as a megarectum. The term megarectum is also used to describe large rectal masses detected on rectal examinations.

This report presents an extremely rare surgical case of postoperative megarectum in an adult patient with an imperforate anus.

## 2. Case Report

**Figure 1:**
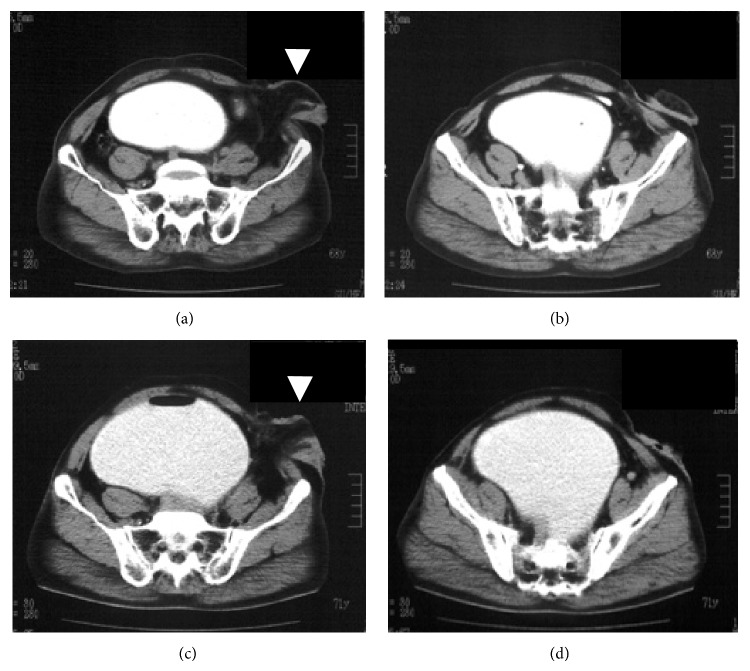
Computed tomography (CT) revealed a large cystic mass in the pelvic cavity at our surgical outpatient clinic ((c), (d)) and at another hospital two years previously ((a), (b)). A sigmoid divided colostomy was created in the left lower abdomen (arrow head).

**Figure 2:**
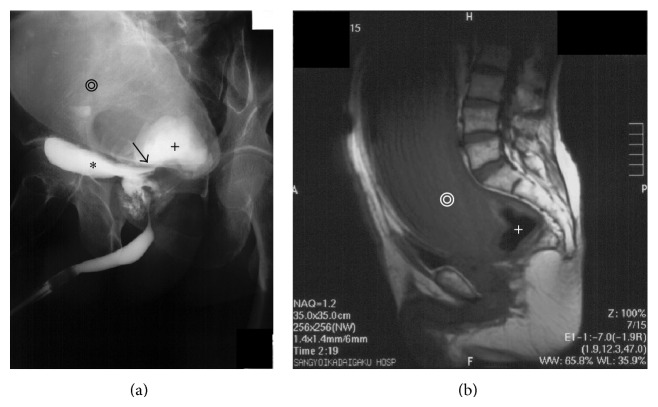
(a) Retrograde urethrography indicated a dilated rectum (black double concentric circles), urinary bladder (black star), and rectourethral fistula between the prostate urethra and lower rectum (black arrow) with stones (black cross). (b) Magnetic resonance imaging (MRI) revealed a megarectum (white double open circles) with stones (white cross).

A 71-year-old Japanese male presented with an increasing lower abdominal mass and frequent urination. He had undergone sigmoid loop colostomy for an imperforate anus as a newborn. At 28 years of age, he underwent an operation for which the details are unclear. At that time, the sigmoid loop colostomy was changed to sigmoid divided colostomy in the left lower abdomen. The patient had not used the colostomy pouch before arriving at our hospital. He was referred to the surgical outpatient clinic for a detailed examination and surgical treatment of an increasing lower abdominal mass, edema of the right lower extremity, and frequent urination. Computed tomography (CT) revealed a large cystic mass measuring 15 × 12 × 8 cm in diameter in the lower abdomen (Figures 1(c) and 1(d)). CT performed two years previously at the other hospital showed a smaller cystic mass measuring 11 cm × 8 cm in diameter in the lower abdomen (Figures 1(a) and 1(b)). Retrograde urethrography indicated a rectourethral fistula and megarectum with stones (Figure 2(a)). Moreover, magnetic resonance imaging (MRI) revealed a megarectum with stones (Figure 2(b)).

A physical examination revealed a mass in the lower abdomen which was 15 × 12 × 8 cm in diameter and edema of the right lower extremity. A sigmoid colostomy incision was created in the patient's left lower abdomen. Laboratory investigations revealed a white blood cell count of 6,700/mm^3^, a hemoglobin level of 13.9 g/dL with a hematocrit value of 40.9%, and a platelet count of 205,000/mm^3^. The renal and liver function test results were all within the normal limits. Coagulation studies revealed prothrombin time of 12.3 seconds and activated partial thromboplastin time of 25.6 seconds.

A small laparotomy incision was created in the right lower abdomen, and a wall of the megarectum was identified. Approximately 2,300 mL of gray muddy fluid was identified and drained. A mucous fistula of the upper rectum was created in the right lower abdomen.

**Figure 3:**
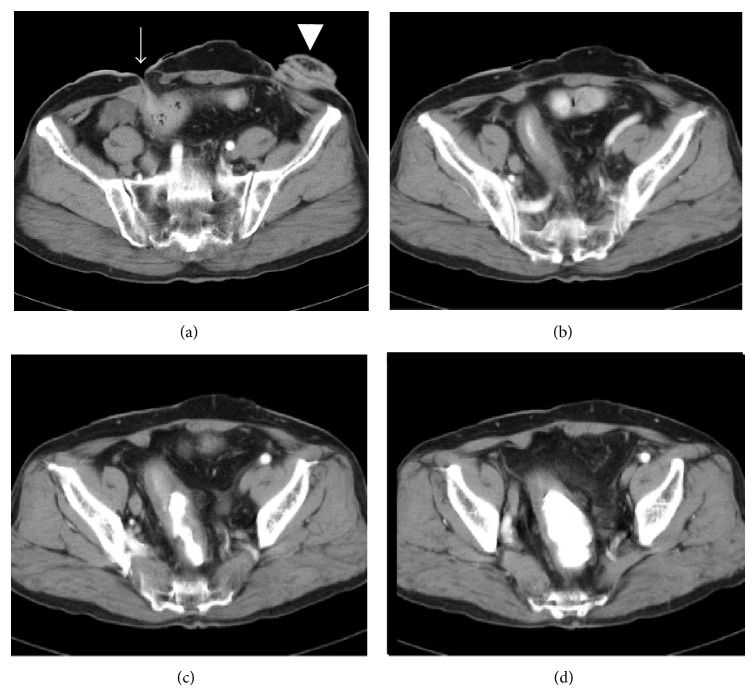
CT performed after surgery indicated that the rectal dilation had resolved ((a), (b), (c), and (d)). A mucous fistula of the upper rectum was created in the right lower abdomen (arrow). A sigmoid divided colostomy was created in the left lower abdomen (arrow head).

The patient had an uneventful recovery. He was discharged from the hospital on the fourteenth day after the operation and was followed up in the outpatient clinic without recurrence of the lower abdominal mass (Figure 3), edema of the right lower extremity, or frequent urination.

## 3. Discussion

Imperforate anus/anorectal malformations are classified into three main groups high (supralevator), intermediate, and low (translevator) according to the relationship between the end of the bowel and pelvic floor [3]. In addition, high and intermediate malformations are subdivided into those with and without fistulas [3]. In the present case, a retrograde urethrography indicated a rectourethral fistula between the prostate urethra and lower rectum, and the distal end of the rectum was located beyond the pelvic floor (Figure 2(a)). Therefore, the patient was diagnosed with a high imperforate anus and rectourethral fistula.

In the present case, the diameter of the expanded rectum was 15 × 12 cm, and the rectum contained 2,300 mL of gray muddy fluid. Therefore, the patient was diagnosed with megarectum. Megarectum is caused by several factors, including severe constipation [6], Hirschsprung's disease, anorectal obstruction, and disorders of the endocrine and central nervous systems [7]. Idiopathic megarectum is an unusual cause of intractable constipation [8, 9], presenting with a chronically dilated rectum alone or in combination with the colon with a morphologically normal small intestine. However, the cause of megarectum in the present case has not been previously reported. We speculate that the cause of the megarectum in this case reflected closure of the anal stump of the sigmoid loop colostomy and rectourethral fistula. The semiclosed cavity of the rectum between the rectosigmoid region and anus may have resulted from the previous operation and then was gradually dilated by the urine due to the rectourethral fistula between the prostate urethra and lower rectum.

Surgical cases of imperforate anus with rectourethral fistulas in adulthood are extremely rare [10, 11]. Odaka et al. reported an unusual case of anorectal agenesis with a rectourethral fistula in a 48-years-old male who was treated with end colostomy and resection of the rectourethral fistula, dilated rectum, and sigmoid colon [10]. Resection of the megarectum and repair of the rectourethral fistula could have been performed in this case. However, a mucous fistula of the upper rectum was created in the right lower abdomen because the patient rejected major surgery.

In conclusion, this report presented a rare surgical case of postoperative megarectum in an adult patient with an imperforate anus and rectourethral fistula.
